# Cortisol secretion after adrenocorticotrophin (ACTH) and Dexamethasone tests in healthy female and male dogs

**DOI:** 10.1186/1751-0147-51-33

**Published:** 2009-08-17

**Authors:** Paula Pessina, Andrea Fernández-Foren, Enrique Cueto, Luis Delucchi, Victor Castillo, Ana Meikle

**Affiliations:** 1 Laboratorio de Técnicas Nucleares, Facultad de Veterinaria, Montevideo, Uruguay; 2 Clínica de Pequeños Animales, Facultad de Veterinaria, Lasplaces 1550 Montevideo, Uruguay; 3 Area de Clínica Médica de Pequeños Animales, Hospital Escuela, U. Endocrinología Universidad de Buenos Aires, Argentina

## Abstract

**Background:**

For the conclusive diagnosis of Cushing's Syndrome, a stimulating ACTH test or a low suppressive Dexamethasone test is used. Reports in other species than the dog indicate that plasma cortisol concentration after ACTH administration is affected by gender. We investigated the effect of gender on the cortisol response to ACTH and Dexamethasone tests in dogs.

**Methods:**

Seven healthy adult Cocker Spaniels (4 females and 3 males) were assigned to a two by two factorial design: 4 dogs (2 females and 2 males) received IV Dexamethasone 0.01 mg/kg, while the other 3 dogs received an IV saline solution (control group). Two weeks later the treatments were reversed. After one month, ACTH was given IV (250 μg/animal) to 4 dogs (2 female and 2 males) while the rest was treated with saline solution (control group). Cortisol concentrations were determined by a direct solid-phase radioimmunoassay and cholesterol and triglycerides by commercial kits.

**Results and Discussion:**

No effect of treatment was observed in metabolite concentrations, but females presented higher cholesterol concentrations. ACTH-treated dogs showed an increase in cortisol levels in the first hour after sampling until 3 hours post injection. Cortisol concentrations in Dexamethasone-treated dogs decreased one hour post injection and remained low for 3 hours, thereafter cortisol concentrations increased. The increase in cortisol levels from one to two hours post ACTH injection was significantly higher in females than males. In Dexamethasone-treated males cortisol levels decreased one hour post injection up to 3 hours; in females the decrease was more pronounced and prolonged, up to 5 hours post injection.

**Conclusion:**

We have demonstrated that cortisol response to ACTH and Dexamethasone treatment in dogs differs according to sex.

## Background

Cushing's Syndrome (CS) is a disorder associated with excessive glucocorticoid production. The CS is one of the most common endocrine pathologies in dogs [1,2]. The excess of cortisol affects different tissues and metabolic pathways, such as the carbohydrate and lipid pathways [3-6]. As a result dogs show glucose intolerance, hyperglycemia and hyperlipemia accompanied with an increase in triglycerides and total cholesterol, [4,7,8] causing signs as polyuria, polydipsia, polyphagia and abdominal enlargement. All characteristics of Cushing's Syndrome [9,10].

Even though it is possible to diagnose CS by clinical signs, laboratory tests and radiology/ecography exams, the syndrome should be confirmed by hormone determination [10]. Determination of basal cortisol is of no meaning, levels are very variable and affected by the stress of sampling. Thus, for the conclusive diagnosis a stimulating ACTH test or a low suppressive Dexamethasone test is used [11]. The suggested hours for sampling after ACTH administration are 30 to 60 min post-injection and for the low dose Dexamethasone test 3 to 4 and 8 hours after the injection [10].

Many laboratories have established reliable reference values for cortisol concentrations in blood of clinically normal animals. However, non pathologic factors that affect adrenocortical secretion may lead to misinterpretation of test results. For other species (humans, ruminants) a gender effect on cortisol concentrations exists [12], but in dogs no sex effect has been found [13]. In sheep, plasma cortisol concentrations after ACTH administration were higher in females than in males [14]. Cortisol affects carbohydrate and lipid metabolism, and even if it is known that ACTH/Dexamethasone administration affects cortisol levels, we found no reports on their effects on cholesterol and triglycerides levels in dogs.

The aim of this study was to determine the effect of ACTH and the low-dose Dexamethasone test on cortisol concentrations determined hourly in healthy male and female dogs. We also investigated the effects of these tests on cholesterol and triglycerides levels.

## Materials and methods

### Animals and blood sampling

Seven healthy adult Cocker Spaniels, 6 to 9 years of age (3 males and 4 females) were used. The mean (± SEM) weight of these animals was 9 ± 0.7 kg. Animals were fed with a commercial diet (Excelent, Purina, Nestlé, Buenos Aires, Argentina) at 20:00 hours. Animal experimentation was performed in compliance with regulations set by the Veterinary Faculty, University of Uruguay, Uruguay.

Animal management and blood sampling was the same in both experimental designs. Animals were catheterized in the cephalic vein at 6:00 and blood samples were taken every hour from 7:00 until 17:00 hours in heparinized vials. Infusions of ACTH, Dexamethasone or saline solutions were given at 9:00 (e.g. two hours after the initiation of the blood sampling). Blood samples were centrifuged at 3000 rpm for 15 min and plasma was stored at -20°C until assayed.

### Experimental Design 1: Low-dose Dexamethasone test

A cross-over design was performed: Dexamethasone 0.01 mg/kg (Dispert S.A., Montevideo, Uruguay) was given IV to four dogs (2 females and 2 males), while the other 3 dogs received IV saline solution. Two weeks later, the Dexamethasone-treated animals received the IV saline solution, while the previous control dogs were treated with the same Dexamethasone dose.

### Experimental Design 2: ACTH test

ACTH was given IV at a dose of 250 μg/animal (Novartis Farmaceutica LTD, England) to 4 dogs (2 females and 2 males) while the rest received saline solution (control group).

#### Hormone determination

Plasma samples were assayed in the Laboratory of Nuclear Techniques, Veterinary Faculty, Montevideo, Uruguay.

Cortisol concentrations were determined by a direct solid-phase radioimmunoassay using DPC kits (Diagnostic Products Corporation, Los Angeles, CA, USA). The analytical detection limit of the assay was 9.6 nmol/L. The intra-assay coefficients of variation for low (27.6 nmol/L) and medium controls (138.0 nmol/L) were 3.4% and 5.3%, respectively. The inter-assay coefficients of variation for the same controls were 8.7% and 7.6%, respectively.

#### Metabolite determination

The methods and commercial kits used were: Cholesterol by enzymatic AA method (Wiener Lab 1220114, Rosario-Argentina) and Triglycerides by GPO/PAP AA Wiener Lab 1780112, Rosario-Argentina). The standatrol was used as an internal control (Wiener Lab 1937553, Rosario-Argentina). The intra-assay coefficient of variation was below 10%.

### Statistical Analyses

Cortisol, cholesterol and triglycerides concentrations were analyzed by the mixed procedure (PROC MIXED de SAS^®^, Statistical Analysis System, SAS Institute Inc., Cary, NC, USA 2000). The statistical model included the effects of treatment, gender and hour of sampling and their interactions. Data are presented in graphs as least square means ± pooled standard error. Significance was considered when P ≤ 0.05, and values between P > 0.05 and P < 0.1 were considered a trend.

## Results

### Test effect

**Figure 1:**
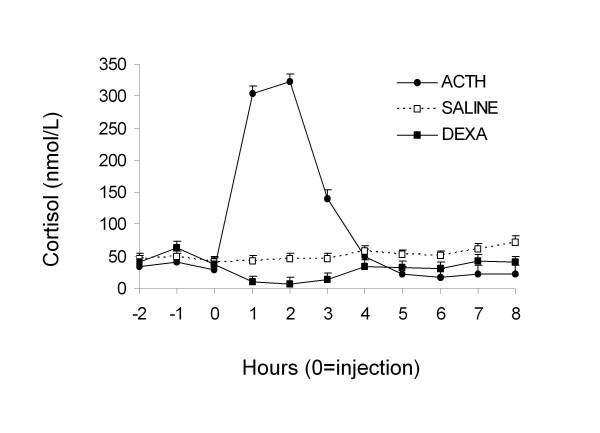
**Cortisol concentrations after ACTH, Dexamethasone and Saline solution treatments in healthy Cocker Spaniel dogs**.

**Figure 2:**
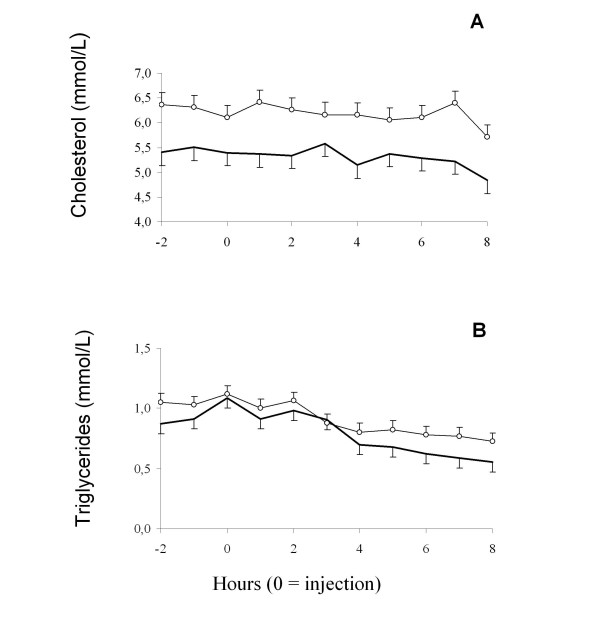
**Cholesterol (A) and triglycerides (B) concentrations in healthy female and male Cocker Spaniel dogs**. Open dots: females. Solid line: males.

Sampling time, treatment and their interaction had a significant effect on cortisol concentrations (P < 0.0001 for all effects). Cortisol concentrations in Dexamethasone treated dogs decreased one hour post injection and remained low for 3 hours, thereafter cortisol concentrations increased. ACTH-test dogs showed an increase in cortisol levels in the first hour after sampling until 3 hours post injection. No differences from basal levels were found 4 hours post injection (Figure 1). No effect of time of sampling on cortisol levels were observed in control dogs (Figure 1). No effect of sampling on cholesterol levels were observed, but triglyceride concentrations decreased at the end of the sampling period (Figure 2).

### Gender effect

**Figure 3:**
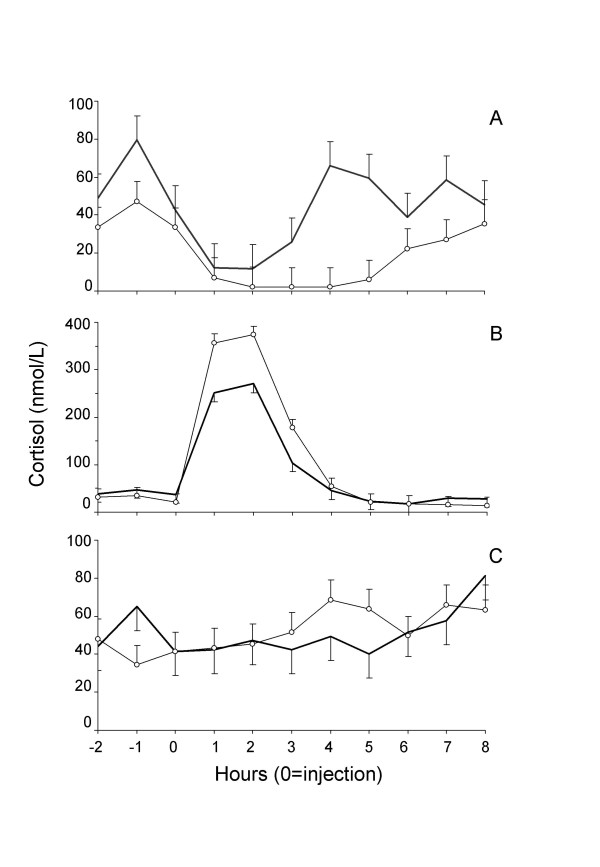
**Cortisol concentrations in female and male healthy Cocker Spaniel dogs after Dexamethasone (A), ACTH (B) and Saline solution (C) treatments**. Observe that the scale in B panel is different from A and C panels. Open dots: females. Solid line: males.

A significant interaction between sex*treatment (P < 0.0001) and sex*treatment*time of sampling (P = 0.0095) on cortisol concentrations were found. In the Dexamethasone-treated males cortisol levels decreased one hour post injection and returned to pre-treatment levels 3 hours later. In the females the decrease was more pronounced and prolonged, returning to pre-treatment levels 6 hours post injection (Figure 3A). This resulted in that cortisol levels were lower in females 4 and 5 hours after Dexamethasone injection. The increase in cortisol levels from one to two hours post ACTH injection was significantly higher in females than males (P < 0.001) (Figure 3B). Control dogs did not show differences among gender (Figure 3C).

There was an effect of gender on cholesterol and triglycerides concentrations, as females had higher cholesterol levels (6.2 ± 0.26 vs 5.2 ± 0.26 mmol/L, P < 0.0001) and triglycerides concentrations (0.91 ± 0.08 vs 0.80 ± 0.07 mmol/L respectively). Since there was no effect of treatment or sampling time on metabolite concentrations, data was pooled according to gender (Figure 2).

## Discussion

A circadian rhythm for cortisol secretion has been reported for humans, monkeys and rats, but this is contradictory in dogs [15-17]. In the present study, cortisol concentrations were measured hourly for 10 hours and no variations were observed. No changes were found in cholesterol concentrations, but triglycerides levels decreased at the end of the experimental period probably due to the prolonged time from the last meal (17 hours after intake).

No effect of treatment was observed in cortisol concentrations. Dexamethasone-treated dogs presented a decrease in the first hour post injection that lasted until 3 hours post injection. Feldman and Nelson [18] found a similar suppression of cortisol secretion after Dexamethasone treatment in dogs sampled hourly, although the levels remained low up to 8 hours post injection. This difference cannot be attributed to the dose, but it could be due to the adjuvant and/or management of the animals (e.g., breed, hours of fasting). Cortisol response to stimulation with ACTH and inhibition with Dexamethasone was similar to other reports considering not only the maximum cortisol concentrations after treatment, but also the duration of the response [18-20]. In the present study, ACTH-treated dogs maintained high cortisol levels up to 3 hours post injection; while Hansen et al (1994)[19] -sampling every 30 min- found a significant increase, similar to ours, in the first 30 min post-injection and a maximum concentration 1.5 hours after injection. Their sampling ended at 2.5 hours and cortisol concentrations were - although lower than 1.5 hour post injection- still different from baseline.

Although our study used a small number of animals, it should be noted that the frequent sampling and cortisol concentrations could not be attributed to the stress of the sampling procedure. We did not find sex differences in basal cortisol levels, which is in agreement with Reimers et al. 1990 [13] who did not find any effect of gender on cortisol concentrations in healthy dogs. On the other hand, the concentration of the precursor of cortisol - cholesterol - was higher in female dogs than males (6.2 vs 5.2 mmol/L), in agreement with Barrie et al 1993 [21].

This study showed that gender affected the cortisol response of the animals when stimulated with ACTH or inhibited with Dexamethasone.

Dexamethasone inhibition of cortisol secretion differed according to sex, as inhibition lasted longer in females than in males. We have not found any reports on different response by gender in Dexamethasone-treated dogs. In humans, the glucocorticoid sensitivity measured by a Dexamethasone suppression test and the combined Dexamethasone suppression/CRH stimulation (Dex-CRH) test used to evaluate the degree of HPA-axis dysregulation in patients with unipolar depression have been shown to be affected by sex [22]. Moreover, in rats, estrogen treatment reduced type I receptors in the anterior pituitary by 50-60%; this down-regulatory effect was seen only in female rats and no change was found for males suggesting that the regulation was steroid independent [23]. Whether the prolonged Dexamethasone inhibition on cortisol secretion in dogs is due to differential pituitary/hypothalamus sensitivity to glucocorticoids remains to be elucidated.

ACTH response - in terms of cortisol secretion - was also affected by gender as females presented higher cortisol concentrations than males. This is in agreement with reports in other species (rat [24,25]; sheep *in vitro *[26]; sheep *in vivo *[27]). Since the treatment was with exogenous ACTH, we can suggest that the peripheral response of the adrenal glands to ACTH has a different regulation among sexes. We have found no reports on different sensitivity of the adrenal gland to ACTH in dogs. On the other hand, we have previously shown the existence of estrogen receptor alpha (ERα) in the sheep adrenal gland and found a varying sensitivity to oestrogens as the ER levels differed according to sex and gonadal status, with the ewes having higher ER levels than rams [27]. These findings indicate that oestrogens most likely affect steroidogenesis directly at the adrenal cortex and suggest that oestrogens are partly responsible for the sex differences in cortisol secretion in sheep. Possible mechanisms by which oestrogens might affect adrenal steroidogenesis have been suggested: influence on adrenocortical sensitivity to ACTH [28,29]; stimulatory effect on the induction of enzymes in the synthesis of glucocorticoids [30]; and/or increase in availability of steroid precursor (cholesterol) by affecting the steroidogenic acute regulatory (StAR) protein [31,32]. In this study the plasma concentrations of the steroid precursor - cholesterol - in female dogs was higher than in males (Figure 2A), as previously shown in the Beagle breed [33]. This finding suggests that the higher cortisol levels found in females after ACTH test is due -at least partially- to a higher availability of its precursor: cholesterol. We have not found any studies on the mechanisms by which oestrogens participate in the regulation of adrenal cortex functions, e.g. cortisol secretion, in dogs.

## Conclusion

In summary, we have demonstrated that the cortisol response to ACTH and Dexamethasone treatment in dogs differs according to sex.

## Abbreviations

ACTH: adrecorticotropin; CS: Cushing's Syndrome.

## Competing interests

The authors declare that they have no competing interests.

## Authors' contributions

PP lead the experimental designs and drafted the manuscript. AFF carried out the inmunoassays and metabolites assays. EC and LD contributed with the experimental designs. VC contributed with the interpretation of the data and correction of the manuscript. AM performed the statistical análisis and helped to draft the manuscript. All authors read and approved the final manuscript.
